# Gender Differences in Body Composition in Pre-Frail Older Adults With Diabetes Mellitus

**DOI:** 10.3389/fendo.2022.795594

**Published:** 2022-02-15

**Authors:** Reshma Aziz Merchant, John Tshon Yit Soong, John E. Morley

**Affiliations:** ^1^ Geriatric Medicine, Department of Medicine, National University Hospital, Singapore, Singapore; ^2^ Department of Medicine, Yong Loo Lin School of Medicine, National University Singapore, Singapore, Singapore; ^3^ Advanced Internal Medicine, Department of Medicine, National University Hospital, Singapore, Singapore; ^4^ Division of Geriatric Medicine, Saint Louis University School of Medicine, St Louis, MO, United States

**Keywords:** pre-frailty, FRAIL scale, diabetes mellitus, body composition, physical function, cognitive function

## Abstract

**Background & Aims:**

Ageing is a risk factor for diabetes mellitus (DM) and frailty. It is associated with body composition changes including increase in fat mass (FM), central fat distribution, decrease in fat free mass (FFM) and skeletal muscle which are risk factors for DM. This study aims to evaluate gender differences in body composition in pre-frail diabetics and association with physical performance, cognitive function and perceived health. In addition, we aim to explore the association of obesity, sarcopenia, sarcopenic obesity, and body composition in pre-frail older adults to DM status.

**Methods:**

Cross-sectional study of 192 pre-frail community dwelling older adults (≥ 65 years). Data was collected on demographics, physical function, cognition, frailty, sarcopenia, perceived health and body composition using the InBody S10. Univariate and multivariate logistic regression were undertaken to explore the association of sarcopenic obesity, obesity, sarcopenia and body composition measures to DM status.

**Results:**

There were insignificant within-gender differences for physical function, cognition and body composition, except for a higher prevalence of obesity defined by body mass index (BMI) and body fat percentage (BF%), increased fat mass index(FMI) and fat free mass index(FFMI) in females with DM. There were significant between-gender differences for those with DM where females overall had lower education levels, lower perceived health, higher prevalence of depression and low mental vitality, lower overall physical function (low short physical performance battery scores, low gait speed and hand grip strength), lower cognitive scores, lower muscle mass and muscle quality with higher FMI, FM/FFM and visceral fat area(VFA). BMI, VFA>100 cm^2^, FMI and FFMI were found to be independently associated with DM status after multivariable adjustment.

**Conclusion:**

Within pre-frail DM vs non-DM, there were insignificant differences in body composition, physical function, cognition and perceived health within gender except for FMI, BF% and FFMI in females. There were significant differences between gender in pre-frail DM in muscle mass, quality, functional, cognitive and mental status. Further longitudinal studies are required to understand the pathogenesis, trajectory of DM and protective role of oral hypoglycemics in pre-frail older adults.

## Introduction

Population ageing is a global phenomenon and the number of older people ≥ 65 years is projected to double to 1.5 billion in 2050 (1). Ageing is a risk factor for chronic diseases, and associated with increased prevalence of frailty, cognitive impairment, and obesity. Ageing is also associated with body composition changes including increase in fat mass (FM), ectopic fat distribution and visceral fat area (VFA), with decrease in fat free mass (FFM) and skeletal muscle which are all precursors of metabolic diseases including diabetes (2–5). Diabetes and its related complications including frailty, cognitive impairment and disability imposes a major threat to health and social care system worldwide. The International Diabetes Federation in 2019 estimated that 19.3% ≥ 65 years old worldwide had diabetes (6). A local study published in 2017 similarly showed the prevalence of diabetes was 23.8% amongst ≥ 65 years old (7).

Ageing is associated with impaired glucose tolerance, increased prevalence of postprandial hyperglycemia and type 2 diabetes mellitus (DM). Physical inactivity, beta-cell dysfunction, decrease in skeletal muscle mass and increase in FM including visceral fat which has pro-inflammatory activity are thought to be the main predisposing factors for DM (8, 9). BMI is often used as a predictor for DM, but it is becoming increasingly evident that BMI is not reliable especially in older adults and needs to be interpreted with caution as loss of physiological height with ageing may lead to over-interpretation (3, 10, 11). Jo et al. showed that 64% of population ≥ 40 years old with normal BMI had a high body fat percentage (BF%), and prevalence of abnormal blood glucose was significantly higher in the normal BMI with high BF% compared with overweight and low BF% (12). BMI is a composite of FM and FFM and high BMI in older adults has been associated with better physical and cognitive function (13, 14). Recently body composition indicators such as waist-to-hip ratio, BF%, body fat mass and VFA have been found to be better predictors of type 2 DM (15, 16).

In recent years, frailty, pre-frailty, and sarcopenia have been found to be associated with increased adverse outcomes in older adults irrespective of underlying comorbidities. Frailty is a multidimensional concept and dynamic. It is defined as a state of decreased physiological reserve which increases vulnerability to adverse outcomes when exposed to stressors (17). Sarcopenia is a common cause of physical frailty and defined as age-related loss in muscle mass and strength with impact on physical performance (18). The prevalence of frailty amongst older adults with diabetes is almost double that of normal population, both share similar pathophysiology and frailty is a major factor associated with death and disability in diabetics and metabolic syndrome (19–22). The prevalence of frailty and pre-frailty worldwide is between 50-60% and both are independent predictors of type 2 diabetes mellitus (7, 17, 23–25). While it is known that diabetics have lower muscle mass and high BF%, there is no data on body composition in pre-frail older adults with and without diabetes mellitus (26).

This study aims to evaluate gender differences in body composition in pre-frail diabetics and association with physical performance, cognitive function and perceived health. In addition, we aim to explore the association of obesity, sarcopenia, sarcopenic obesity, and body composition in pre-frail older adults to DM status.

## Materials and Methods

This was a cross-sectional analysis of 192 pre-frail community dwelling older adults recruited for multidomain intervention from primary and community care in Singapore.

### Demographics and Covariates

Trained research staff administered questionnaires covering demographics, chronic diseases, medications, falls, function, cognition, frailty, sarcopenia, depression, and perceived health. Multimorbidity was defined as ≥ 2 self-reported chronic conditions. Mini nutritional Assessment tool was used to assess malnutrition risk (27). Activities of daily living (ADL) was assessed using Katz activity of daily living and instrumental activities of daily living (IADL) using Lawton’s IADL scale (28, 29). Frailty was assessed using the FRAIL scale (Fatigue, Resistance, Aerobic, Illness, and Loss of Weight), where pre-frail was defined based on scores of 1-2 (30). SARC-F was used to screen for sarcopenia (lifting and carrying 10 pounds, walking across a room, transferring from bed/chair, climbing a flight of 10 stairs, and frequency of falls in the past 1 year) with a range of 0-10 (31). Cognitive status was assessed using the Montreal Cognitive Assessment (MoCA) (32). The Rapid Assessment of Physical Activity (RAPA) tool was used to assess physical activity (33). Physically active was based on WHO recommendations of ≥ 150 minutes of moderate intensity, or ≥ 75 minutes of vigorous intensity aerobic physical activity per week (34). The 15-item Geriatric Depression Scale (GDS) was used to screen for depression with ≥ 5 was used to define depression (35). Three questions from the GDS were used to define mental vitality: 1) Are you basically satisfied with your life? 2) Do you feel that your life is empty? 3) Do you feel full of energy? A score of zero defined high mental vitality while a score between one to three signified low mental vitality (36). EuroQol vertical visual analogue scale (EQ-VAS) was used to evaluate perceived health (37). DM status was self-reported by means of questionnaire.

### Physical Function, Waist Circumference and Calf Circumference

Maximum handgrip strength (HGS), gait speed (GS) and Short Physical Performance Battery test (SPPB) was measured. Calf circumference (CC) was measured in seated position with feet on the floor and leg positioned at 90° using non-elastic tape and taken at maximal circumference. HGS of the dominant hand was measured using Jamar hand dynamometer in the seated position with elbow flexed at 90°. Waist circumference (WC) was measured midpoint between the last rib and iliac crest. The SPPB included 3 components on balance, 4m gait speed and chair stand with a maximum score of 12 points (4 points per-component).

### Body Composition and Body Mass Index

BMI was calculated by dividing body weight (kg) by height squared (m^2^). Cut offs of 23 kg/m^2^ and 27.5 kg/m^2^ were used to define overweight and obesity respectively based on WHO recommendations for Asians (38). Underweight older adults defined as having a BMI of <18.5 kg/m^2^ were excluded. InBody S10, a multi-frequency bioelectrical impedance analyzer was used to estimate body composition including FM, FFM, body cell mass, appendicular skeletal muscle (ASM) and whole-body phase angle. Fat Mass Index (FMI) and Fat Free Mass Index (FFMI) were calculated as FM divided by height squared and FFM divided by height squared, respectively. Obesity was defined by BF% >25% in males and >35% in females (39). Normal muscle mass was defined based on to the 2019 AWGS criteria with ASM/height² cut-offs of ≥ 7.0 kg/m^2^ for males and ≥ 5.7 kg/m^2^ for females (40). Sarcopenia diagnosis was based on the 2019 AWGS criteria of gender specific cut offs for ASM/height² and either low HGS (<28kg for male and <18kg for female) or slow GS of <1m/s. Sarcopenic obesity was defined as having an ASM to BMI ratio of < 0.789 and BF% > 25% in males or an ASM to BMI ratio of <0.512 & BF% > 55 in females (39). Muscle quality was defined based on HGS/ASMBMI (41).

### Statistical Analysis

Within and between gender differences of the included participants for demographics, co-morbidity, anthropometrics, clinical assessments and bioimpedance analysis by DM status are represented in a frequency table. Categorical variables are presented as frequencies with percentages, while continuous variables are presented as mean with standard deviation. Significance testing by Chi-Square test for categorical and one-way ANOVA for continuous variables are presented.

Univariate and multivariate logistic regression was undertaken to explore the association of sarcopenic obesity, obesity, sarcopenia definitions, as well as body composition with DM status. Adjustment for weight loss, physical activity, gender, ethnicity, and age was undertaken for the multivariate regression. Odds ratios with confidence intervals, sensitivity, and specificity at Youden point and Area under the Receiver Operator Characteristic (ROC) Curve are presented. Statistical analysis was conducted using R Package version 4.1.0.

### Ethics Approval and Informed Consent

The study conformed to the principles of the Declaration of Helsinki and was approved by The National Healthcare Group Domain Specific Review Board (Reference: 2017/00035 and 2018/01183). Informed consent was obtained from all participants.

## Results

Of the 192 pre-frail participants included in this study, 82(42.7%) were male and 110(57.3%) female, with a mean age of 73 years ± 5.7 years. The predominant ethnic group was Chinese (82.8%) and average number of years in formal education was 7.8 ± 4.3 years.

Within gender differences of participant characteristics are displayed in **Table 1**. In pre-frail older participants, BMI and WC was significantly higher in females with DM compared to non-diabetics (mean BMI of 27.3 ± 4.3 versus 25.2 ± 3.8; p=0.008 and mean WC of 96.2 ± 10.7cm versus 89.8 ± 12.2cm; p=0.038 respectively). This difference was not found between male diabetics and non-diabetics. Both male and female participants with DM had significantly increased multimorbidity compared to those with non-diabetics, with female participants with DM having significantly increased levels of hypertension and hyperlipidaemia compared to non-diabetics. Bioimpedance analysis revealed significant increases in FMI (mean 10.8 ± 3.3 versus 9.3 ± 3.3) and FFMI (mean 16.5 ± 1.7 versus 15.8 ± 1.7) in females with DM compared to non-diabetics. The prevalence of obesity defined by BF% was significantly higher in females with DM compared with non-diabetics, 71.2% and 50.0% respectively. This difference was not found between male diabetics and non-diabetics.

**Table 1:** Characteristics table displaying within gender differences by Diabetes Mellitus (DM) Status.

	All (n=192)	Male 82 (42.7%)			Female 110 (57.3%)		
		No DM 36 (43.9%)	DM 46 (56.1%)	P value	No DM 58 (52.7%)	DM 52 (47.3%)	P value
**Demographics**							
Age	73.04 ± 5.73	74.39 ± 6.74	72.59 ± 5.41	0.183	72.52 ± 5.50	73.08 ± 5.52	0.596
Ethnicity				0.137			0.636
Chinese	159 (82.8)	32 (88.9)	33 (71.7)		50 (86.2)	44 (84.6)	
Malay	13 (6.8)	2 (5.6)	4 (8.7)		4 (6.9)	3 (5.8)	
Indian	19 (9.9)	2 (5.6)	9 (19.6)		3 (5.2)	5 (9.6)	
Others	1 (0.5)				1 (1.7)	0 (0.0)	
BMI (kg/m^2^)	25.89 ± 4.17	24.69 ± 3.52	26.00 ± 4.57	0.161	25.24 ± 3.76	27.33 ± 4.33	**0.008**
BMI status				0.895			**0.019**
Normal	50 (26.0)	13 (36.1)	11 (23.9)		16 (27.6)	10 (19.2)	
Overweight	61 (31.8)	8 (22.2)	13 (28.3)		14 (24.1)	26 (50.0)	
Obese	81 (42.2)	15 (41.7)	22 (47.8)		28 (48.3)	16 (30.8)	
Depression	59 (31.4)	9 (25.0)	8 (17.4)	0.569	23 (41.1)	19 (38.0)	0.901
Hyperlipidaemia	157 (81.8)	29 (80.6)	42 (91.3)	0.275	38 (65.5)	48 (92.3)	**0.002**
Hypertension	139 (72.8)	29 (80.6)	37 (80.4)	1	33 (56.9)	40 (78.4)	**0.029**
Multimorbidity	161 (83.9)	29 (80.6)	45 (97.8)	**0.025**	36 (62.1)	51 (98.1)	**<0.001**
Education (years)	7.84 ± 4.27	8.26 ± 4.28	9.39 ± 3.88	0.216	7.58 ± 4.48	6.40 ± 3.93	0.153
Calf circumference (cm)	35.85 ± 4.51	36.17 ± 3.55	36.44 ± 3.12	0.728	34.93 ± 3.69	36.17 ± 6.58	0.23
Waist circumference (cm)	93.05 ± 10.93	92.84 ± 9.32	94.93 ± 9.75	0.449	89.83 ± 12.16	96.18 ± 10.74	**0.038**
Nutritional status (MNA)	21.84 ± 2.10	21.52 ± 2.39	22.17 ± 2.19	0.227	22.22 ± 1.76	21.41 ± 2.06	0.05
≥ 1 ADL impairment	33 (17.2)	4 (11.1)	6 (13.0)	1	13 (22.4)	10 (19.2)	0.861
≥ 1 IADL impairment	57 (29.7)	13 (36.1)	13 (28.3)	0.604	17 (29.3)	14 (26.9)	0.948
Perceived Health (EQ-VAS)	69.35 ± 14.74	70.22 ± 12.96	73.11 ± 13.62	0.336	68.14 ± 16.43	66.73 ± 14.58	0.639
Moderate Physical Activity	78 (40.8)	17 (47.2)	23 (50.0)	0.978	19 (33.3)	19 (36.5)	0.881
Physical activity (RAPA)	3.35 ± 1.61	3.81 ± 1.70	3.70 ± 1.71	0.773	3.07 ± 1.50	3.02 ± 1.49	0.859
MoCA	24.96 ± 4.27	25.94 ± 3.26	25.89 ± 2.75	0.936	24.51 ± 5.10	23.94 ± 4.75	0.551
**Physical Function**							
Sarcopenia (SARC-F)	1.58 ± 1.81	1.28 ± 1.65	0.83 ± 1.22	0.157	1.84 ± 1.89	2.18 ± 2.03	0.376
Sarcopenia (AWGS 2019)	48 (25.0)	8 (22.2)	14 (30.4)	0.561	15 (25.9)	11 (21.2)	0.722
Sarcopenic obesity	58 (30.2)	12 (33.3)	14 (30.4)	0.967	15 (25.9)	17 (32.7)	0.564
≥ 5% weight loss	19 (9.9)	8 (22.2)	5 (10.9)	0.275	4 (6.9)	2 (3.8)	0.777
At least 1 fall in past year	51 (26.6)	7 (19.4)	11 (23.9)	0.829	15 (25.9)	18 (34.6)	0.428
Low mental vitality	107 (56.9)	16 (44.4)	18 (39.1)	0.796	40 (71.4)	33 (66.0)	0.695
Handgrip strength (kg)	22.13 ± 7.20	28.05 ± 6.20	27.40 ± 6.37	0.642	18.72 ± 4.73	17.17 ± 4.40	0.081
Chair time (s)	13.52 ± 5.37	12.55 ± 3.88	11.98 ± 4.27	0.54	14.09 ± 6.38	15.04 ± 5.65	0.439
Chair time ≥ 12s	93 (52.8)	18 (52.9)	17 (38.6)	0.303	26 (51.0)	32 (68.1)	0.13
Gait speed	0.91 ± 0.27	0.96 ± 0.27	0.97 ± 0.27	0.915	0.90 ± 0.29	0.82 ± 0.23	0.111
SPPB score	9.58 ± 2.33	10.06 ± 1.82	10.35 ± 2.45	0.552	9.26 ± 2.36	8.92 ± 2.31	0.453
**Body Composition**							
ASM (kg/m^2^)	7.12 ± 1.75	7.79 ± 1.74	7.82 ± 1.80	0.933	6.56 ± 1.67	6.67 ± 1.43	0.709
Normal muscle mass	135 (70.3)	26 (72.2)	31 (67.4)	0.818	39 (67.2)	39 (75.0)	0.494
Muscle quality ^	32.65 (10.49)	33.89 ± 11.37	35.07 ± 12.17	0.659	32.64 ± 10.30	30.48 ± 8.24	0.233
Phase Angle (50Khz)	4.98 (0.99)	5.04 ± 1.03	5.17 ± 0.97	0.558	4.96 ± 1.13	4.78 ± 0.80	0.353
Obese (by fat mass %)	119 (62.0)	24 (66.7)	29 (63.0)	0.914	29 (50.0)	37 (71.2)	**0.039**
Body fat percentage (BF%)	33.39 ± 8.79	27.48 ± 6.08	28.34 ± 8.30	0.603	36.20 ± 7.81	38.81 ± 6.98	0.069
Fat Mass Index	8.84 ± 3.49	6.92 ± 2.50	7.54 ± 3.40	0.363	9.27 ± 3.25	10.84 ± 3.32	**0.014**
Fat Free Mass Index	16.93 ± 2.04	17.76 ± 1.86	18.19 ± 2.03	0.329	15.78 ± 1.70	16.51 ± 1.67	**0.026**
Fat Mass to Fat Free Mass	0.53 ± 0.21	0.39 ± 0.13	0.42 ± 0.19	0.462	0.59 ± 0.21	0.65 ± 0.19	0.104
Visceral fat area (cm^2^)	101.80 ± 47.32	75.47 ± 27.88	88.43 ± 40.09	0.117	109.26 ± 52.54	124.89 ± 46.99	0.134
Visceral fat area > 100cm^2^	78 (46.2)	4 (12.1)	16 (37.2)	**0.028**	24 (53.3)	34 (70.8)	0.127
Body cell mass	25.69 ± 7.25	29.15 ± 8.28	30.16 ± 6.14	0.527	21.69 ± 5.74	23.73 ± 5.56	0.063

n(%); mean ± SD; significant in bold; sarcopenic obesity definition: Male: ASM : BMI < 0.789 & BF% > 25; Female: ASM : BMI<0.512 & BF%>35.

AWGS, Asian Workgroup for Sarcopenia; BMI, Body Mass Index; ^HGS/ASMBMI; MNA, mini nutritional assessment; Muscle quality ^:HGS/ASMBMI.

Between gender differences of participant characteristics are displayed in **Table 2**. There was significantly lower education and perceived health in females with DM compared to males (6.4 ± 3.9 versus 9.4 ± 3.9 years and EQ-VAS of 66.7 ± 14.6 versus 73.1 ± 13.6 respectively). There were significantly lower Rapid Assessment of Physical Activity scores but no difference in moderate physical activity levels in females compared with males with and without DM. The prevalence of low mental vitality was significantly higher in females compared with males regardless of underlying DM status (non-DM: 71.4% versus 44.4%, DM: 66.0% vs 39.1%). Females with DM had more than twice the prevalence of depression compared with males with DM, 38.0% versus 17.4%. Females had significantly lower MNA scores (21.4 ± 2.1 versus 22.2 ± 2.1). Overall, females compared with males with DM had slower chair times (mean 15s ± 5.7 versus 12s ± 4.3), slower GS (0.8 ± 0.2 m/s versus 1.0 ± 0.3 m/s), lower SPPB scores (8.9 ± 2.3 versus 10.4 ± 2.5) and MoCA scores (23.9 ± 4.8 versus 25.9 ± 2.8). This difference was not found in the non-diabetic population. Bioimpedance analysis revealed significantly lower mean ASM (kg/m2) mass, FFMI and body cell mass, and higher BF%, FMI, FM/FFM and VFA in females compared to males, in both the DM and non-diabetic population. Mean whole body phase angle was significantly lower in females with DM compared to males (4.78 ± 0.80 versus 5.17 ± 0.97. Muscle quality was significantly lower in females with DM, 30.48 ± 8.24 compared with males 35.07 ± 12.17. Body cell mass was lower in females regardless of the DM status.

**Table 2:** Characteristics table displaying between gender differences by Diabetes Mellitus (DM) Status.

	All (n=192)	No DM 94 (49.0%)			DM 98 (51.0%)		
		Male 36 (38.3%)	Female 58 (61.7%)	P value	Male 46 (46.9%)	Female 52 (53.1%)	P value
**Demographics**							
Age	73.04 ± 5.73	74.39 ± 6.74	72.52 ± 5.50	0.145	72.59 ± 5.41	73.08 ± 5.52	0.659
Ethnicity				0.871			0.287
Chinese	159 (82.8)	32 (88.9)	50 (86.2)		33 (71.7)	44 (84.6)	
Malay	13 (6.8)	2 (5.6)	4 (6.9)		4 (8.7)	3 (5.8)	
Indian	19 (9.9)	2 (5.6)	3 (5.2)		9 (19.6)	5 (9.6)	
Others	1 (0.5)	0 (0.0)	1 (1.7)				
BMI (kg/m^2^)	25.89 ± 4.17	24.69 ± 3.52	25.24 ± 3.76	0.483	26.00 ± 4.57	27.33 ± 4.33	0.143
BMI status				0.68			0.083
Normal	50 (26.0)	13 (36.1)	16 (27.6)		11 (23.9)	10 (19.2)	
Overweight	61 (31.8)	8 (22.2)	14 (24.1)		13 (28.3)	26 (50.0)	
Obese	81 (42.2)	15 (41.7)	28 (48.3)		22 (47.8)	16 (30.8)	
Depression	59 (31.4)	9 (25.0)	23 (41.1)	0.175	8 (17.4)	19 (38.0)	**0.044**
Hyperlipidaemia	157 (81.8)	29 (80.6)	38 (65.5)	0.183	42 (91.3)	48 (92.3)	1
Hypertension	139 (72.8)	29 (80.6)	33 (56.9)	**0.033**	37 (80.4)	40 (78.4)	1
Multimorbidity	161 (83.9)	29 (80.6)	36 (62.1)	0.098	45 (97.8)	51 (98.1)	1
Education (years)	7.84 ± 4.27	8.26 ± 4.28	7.58 ± 4.48	0.475	9.39 ± 3.88	6.40 ± 3.93	**<0.001**
Calf circumference (cm)	35.85 ± 4.51	36.17 ± 3.55	34.93 ± 3.69	0.119	36.44 ± 3.12	36.17 ± 6.58	0.805
Waist circumference (cm)	93.05 ± 10.93	92.84 ± 9.32	89.83 ± 12.16	0.312	94.93 ± 9.75	96.18 ± 10.74	0.666
Nutritional status (MNA)	21.84 ± 2.10	21.52 ± 2.39	22.22 ± 1.76	0.145	22.17 ± 2.19	21.41 ± 2.06	0.099
≥ 1 ADL impairment	33 (17.2)	4 (11.1)	13 (22.4)	0.268	6 (13.0)	10 (19.2)	0.58
≥ 1 IADL impairment	57 (29.7)	13 (36.1)	17 (29.3)	0.646	13 (28.3)	14 (26.9)	1
Perceived Health (EQ-VAS)	69.35 ± 14.74	70.22 ± 12.96	68.14 ± 16.43	0.523	73.11 ± 13.62	66.73 ± 14.58	**0.03**
Moderate Physical Activity	78 (40.8)	17 (47.2)	19 (33.3)	0.262	23 (50.0)	19 (36.5)	0.255
Physical activity (RAPA)	3.35 ± 1.61	3.81 ± 1.70	3.07 ± 1.50	**0.031**	3.70 ± 1.71	3.02 ± 1.49	**0.039**
MoCA	24.96 ± 4.27	25.94 ± 3.26	24.51 ± 5.10	0.136	25.89 ± 2.75	23.94 ± 4.75	**0.016**
**Physical Function**							
Sarcopenia (SARC-F)	1.58 ± 1.81	1.28 ± 1.65	1.84 ± 1.89	0.142	0.83 ± 1.22	2.18 ± 2.03	**<0.001**
Sarcopenia (AWGS 2019)	48 (25.0)	8 (22.2)	15 (25.9)	0.879	14 (30.4)	11 (21.2)	0.412
Sarcopenic obesity	58 (30.2)	12 (33.3)	15 (25.9)	0.587	14 (30.4)	17 (32.7)	0.982
≥ 5% weight loss	19 (9.9)	8 (22.2)	4 (6.9)	0.065	5 (10.9)	2 (3.8)	0.34
At least 1 fall in past year	51 (26.6)	7 (19.4)	15 (25.9)	0.643	11 (23.9)	18 (34.6)	0.349
Low mental vitality	107 (56.9)	16 (44.4)	40 (71.4)	**0.018**	18 (39.1)	33 (66.0)	**0.015**
Handgrip strength (kg)	22.13 ± 7.20	28.05 ± 6.20	18.72 ± 4.73	**<0.001**	27.40 ± 6.37	17.17 ± 4.40	**<0.001**
Chair time (s)	13.52 ± 5.37	12.55 ± 3.88	14.09 ± 6.38	0.214	11.98 ± 4.27	15.04 ± 5.65	**0.005**
Chair time ≥ 12s	93 (52.8)	18 (52.9)	26 (51.0)	1	17 (38.6)	32 (68.1)	**0.009**
Gait speed	0.91 ± 0.27	0.96 ± 0.27	0.90 ± 0.29	0.297	0.97 ± 0.27	0.82 ± 0.23	**0.003**
SPPB score	9.58 ± 2.33	10.06 ± 1.82	9.26 ± 2.36	0.087	10.35 ± 2.45	8.92 ± 2.31	**0.004**
**Body Composition**							
ASM (kg/m^2^)	7.12 ± 1.75	7.79 ± 1.74	6.56 ± 1.67	**0.001**	7.82 ± 1.80	6.67 ± 1.43	**0.001**
Normal muscle mass	135 (70.3)	26 (72.2)	39 (67.2)	0.781	31 (67.4)	39 (75.0)	0.543
Muscle quality ^	32.65 (10.49)	33.89 ± 11.37	32.64 ± 10.30	0.587	**35.07** ± **12.17**	**30.48** ± **8.24**	**0.031**
Phase Angle (50Khz)	4.98 (0.99)	5.04 ± 1.03	4.96 ± 1.13	0.716	5.17 ± 0.97	4.78 ± 0.80	**0.031**
Obese (by fat mass %)	119 (62.0)	24 (66.7)	29 (50.0)	0.171	29 (63.0)	37 (71.2)	0.523
Body fat (%)	33.39 ± 8.79	27.48 ± 6.08	36.20 ± 7.81	**<0.001**	28.34 ± 8.30	38.81 ± 6.98	**<0.001**
Fat Mass Index	8.84 ± 3.49	6.92 ± 2.50	9.27 ± 3.25	**<0.001**	7.54 ± 3.40	10.84 ± 3.32	**<0.001**
Fat Free Mass Index	16.93 ± 2.04	17.76 ± 1.86	15.78 ± 1.70	**<0.001**	18.19 ± 2.03	16.51 ± 1.67	**<0.001**
Fat Mass to Fat Free Mass	0.53 ± 0.21	0.39 ± 0.13	0.59 ± 0.21	**<0.001**	0.42 ± 0.19	0.65 ± 0.19	**<0.001**
Visceral fat area (cm^2^)	101.80 ± 47.32	75.47 ± 27.88	109.26 ± 52.54	**0.001**	88.43 ± 40.09	124.89 ± 46.99	**<0.001**
Visceral fat area > 100cm^2^	78 (46.2)	4 (12.1)	24 (53.3)	**<0.001**	16 (37.2)	34 (70.8)	**0.003**
Body cell mass	25.69 ± 7.25	29.15 ± 8.28	21.69 ± 5.74	**<0.001**	30.16 ± 6.14	23.73 ± 5.56	**<0.001**

n(%); mean ± SD; significant in bold; Sarcopenic obesity definition: Male: ASM : BMI < 0.789 & BF% > 25; Female: ASM : BMI<0.512 & BF%>35. AWGS: Asian Workgroup for Sarcopenia BMI, Body Mass Index; ^HGS/ASMBMI; MNA, mini nutritional assessment; Muscle quality ^:HGS/ASMBMI.

The association of sarcopenic obesity, obesity, sarcopenia, and body composition to DM status are displayed in **Table 3**. Both unadjusted and adjusted models for BMI were significantly associated to DM status, with odds ratios of 1.11(95% CI 1.02-1.21);p=0.013 after adjustment for weight loss, physical activity, gender, ethnicity, age, education, EQ-VAS, GDS, mental vitality, physical performance, and MoCA. WC was significantly associated with DM status (odds ratio 1.04 (95% CI 1.00-1.08; p=0.03) but loses significance after adjustment for the variables above. Both unadjusted and adjusted models for FFMI were significantly associated with DM status (odds ratios of 1.21; 95% CI 1.05-1.42; P=0.01 and 1.23(95% CI 1.02-1.49; p=0.03respectively). Adjusted FMI was significantly associated with DM status (odds ratio of 1.11; 95% CI 1.00-1.23; p=0.04).

**Table 3:** Sarcopenic obesity, obesity, sarcopenia definitions and body composition analysis associations with Diabetes Mellitus status.

Variables	Model type	OR (97.5% CI)	Specificity	Sensitivity	AUC
BMI	Adjusted	**1.11(1.02-1.21);p=0.013**	**0.71**	**0.56**	**0.66**
	Unadjusted	**1.11(1.03-1.19);P=0.007**	**0.66**	**0.61**	**0.61**
BMI ≥ 27.5	Adjusted	2.27(0.99-5.33);p=0.05	0.70	0.60	0.66
	Unadjusted	**2.45(1.15-5.34); P=0.02**	**0.76**	**0.40**	**0.59**
Waist Circumference (cm)	Adjusted	1.03(0.99-1.07);p=0.15	0.68	0.69	0.70
	Unadjusted	**1.04(1.00-1.08); P=0.03**	**0.77**	**0.53**	**0.63**
Visceral Fat Area cm^2^	Adjusted	1.01(0.99-1.01);p=0.07	0.62	0.71	0.66
	Unadjusted	1.00(0.99-1.01); P=0.08	0.64	0.58	0.58
Visceral fat area > 100cm^2^	Adjusted	**2.94(1.41-6.38);p=0.005**	**0.69**	**0.76**	**0.54**
	Unadjusted	**2.17(1.17-4.08); P=0.01**	**0.64**	**0.55**	**0.59**
Percentage Body Fat (BF%)	Adjusted	1.02(0.98-1.07);p=0.22	0.79	0.41	0.62
	Unadjusted	1.01(0.98-1.05); P=0.44	0.77	0.42	0.54
Obese (BF% by gender)*	Adjusted	1.36(0.72-2.57);p=0.33	0.59	0.64	0.62
	Unadjusted	1.55(0.87-2.82); P=0.14	0.43	0.67	0.55
Fat Mass Index	Adjusted	**1.11(1.00-1.23);p=0.04**	**0.88**	**0.39**	**0.65**
	Unadjusted	1.07(0.99-1.18); P=0.07	0.74	0.44	0.58
Fat Free Mass Index	Adjusted	**1.23(1.02-1.49);p=0.03**	**0.78**	**0.51**	**0.66**
	Unadjusted	**1.21(1.05-1.42); P=0.01**	**0.42**	**0.81**	**0.61**
Fat Mass to Fat Free Mass	Adjusted	2.94(0.55-17.00);P=0.21	0.84	0.37	0.63
	Unadjusted	1.81(0.48-7.01); P=0.39	0.77	0.42	0.54
Appendicular Skeletal Muscle Mass Index (kg/m2)	Adjusted	1.02(0.84-1.25);p=0.85	0.44	0.77	0.62
	Unadjusted	1.05(0.90-1.26); P=0.50	0.37	0.83	0.57
Muscle Quality^#^	Adjusted	0.99(0.96-1.03) ;p=0.75	0.50	0.73	0.62
	Unadjusted	0.99(0.97-1.02); P=0.76	0.17	0.89	0.51
Sarcopenia^	Adjusted	0.93(0.46-1.88);p=0.84	0.47	0.73	0.62
	Unadjusted	1.04(0.54-2.01); P=0.90	0.75	0.26	0.50
Sarcopenic Obesity^§^	Adjusted	0.91(0.45-1.87);p=0.81	0.49	0.73	0.61
	Unadjusted	1.13(0.61-2.11); P=0.70	0.71	0.32	0.51

Adjustment for weight loss, physical activity, gender, ethnicity, age, education, EQ-VAS, GDS, mental vitality, physical performance, MoCA; Specificity and Sensitivity at Youden point, AUC, Area under receiver operating characteristic curve; significant models in bold. BMI, body mass index.

^§^Sarcopenic obesity definition: Male: ASM : BMI < 0.789 & BF% > 25; Female: ASM : BMI<0.512 & BF%>35; ^#^Muscle Quality: HGS/ASMBMI; Obesity*: BF% > 25% for male and > 35% for female; sarcopenia^: 2019 Asian Workgroup for Sarcopenia definition.

## Discussion

Our study highlights two very significant aspects. Firstly, there were insignificant physical function, cognition, and body composition differences within gender for pre-frail male and female except for higher prevalence of obesity defined by BMI and BF%, increase FMI and FFMI in females with DM. Secondly, there were significant differences between gender for those with DM where females overall had lower education levels, lower perceived health, lower RAPA scores, higher prevalence of depression and low mental vitality, lower overall physical function as shown by low SPPB scores, low GS and HGS, lower MoCA, lower muscle mass, lower muscle quality, body cell mass and whole body phase angle with higher FMI, FM/FFM and VFA. A trend was observed for female with DM to have lower mean MNA scores compared to male. Overall obesity defined by BMI, FFMI, FMI and VFA >100cm^2^ was significantly associated with DM status in pre-frail older adults, and not BF%.

Chronic inflammation is a key element in pre-frailty, sarcopenia, sarcopenic obesity and metabolic diseases such as diabetes (42–44). Body composition changes with ageing especially increase in visceral fat and ectopic fat distribution is pro-inflammatory and obesity is associated among others with high baseline C-reactive protein and interleukin-6 (45). Intramuscular fat infiltration together with sedentary lifestyle, poor dietary habits and effects of medications further aggravates insulin resistance in older adults (46). Obesity is associated with mitochondrial dysfunction, which in is also thought to be responsible for insulin resistance, frailty, and sarcopenia (47–49). Almost 8 out of 10 of females and 7 in 10 males in our study were classified as overweight or obese based on BMI cut-off. In recent years, there has been increasing literature on metabolically healthy obesity (MHO) and metabolically unhealthy obesity (MUO). Increase in FFMI in older obese women has been shown to be an independently associated with MUO which in turn is a predictor of adverse metabolic health (50). Pre-frail females with DM in our study had significantly higher FFMI, FMI, hypertension and hyperlipidaemia prevalence compared with non-diabetics. After adjustment, BMI, FFMI, FMI and VFA >100 cm^2^ were independently associated with DM status further supporting the concept of MUO.

In our study, there were no functional, cognition and body composition differences within gender except for increase in BMI, FMI, FFMI and BF% in females. While muscle quality was overall lower in females, there was no significant differences in those with DM and no DM. There were significant differences in function, cognition, perceived health, depression and body composition between gender in those with DM. Hormonal changes with ageing and menopause such as a rapid fall in oestrogen levels, higher testosterone levels and lower sex hormone-binding globulin are associated with increase in body weight, redistribution of FM, decrease in FFM, insulin resistance and Type 2 DM in women compared to men (51). Within gender, there was a trend for diabetics to have higher ASM(kg/m^2^) and FFMI but significant only for FFMI in females without any significant impact on functional status.

Phase angle is considered as an index of muscle quality and as an indicator of muscle catabolism. It is also a measure of cell vitality and a prognostic marker for frailty, mortality, and functional status (52). Similarly, body cell mass is a measure of metabolic activity, indicator of loss of muscle mass with ageing, nutrition, and inflammation (53). While there was no significant within gender differences for phase angle between diabetics and non-diabetics, pre-frail female with DM exhibited a significantly lower phase angle compared with males. This was further supported by overall lower muscle quality in females with DM. This is in contrary to a recently published study where males with DM had overall lower phase angle (52). Body cell mass was significantly lower in females regardless of their diabetes status which requires further validation.

Diabetes has been associated with low muscle quality, muscle mass and strength which was not shown in our pre-frail participants (54). While the possible explanations include the shared pathophysiology including chronic inflammation, hormonal changes with age and dysfunctional adipose tissue, the effect of medications like metformin was not evaluated. Metformin has immunomodulatory effect and has been associated with lower risk of frailty, lower mortality, lower inflammation in diabetics and protective against sarcopenia (44, 55, 56). While we have no information on metformin use in our study participants, metformin use in patients with DM in a local tertiary centre in 2017 was 59.6%. Similarly, while sarcopenic obesity and DM are closely associated due to shared risk factors, this was not shown in our pre-frail participants (57).

### Methodological Considerations

Our study has several strengths. It represents a snapshot of community dwelling pre-frail older adults in Singapore and to date, there is limited data on pre-frail body composition especially comparing diabetics and non-diabetics. The study utilizers objective measures of muscle strength and robust measures of body composition, together with validated clinical tools to measure physical performance, cognitive function, and perceived health. Our study has several limitations which warrants mention. Absolute consensus for an operational definition for prefrailty has not been achieved, however it is acknowledged in a recent publication that it lies along the frailty continuum (58). However, we utilized a well validated FRAIL score to define the cohort. Due to the cross-sectional study design, lack of information on DM control, duration and treatment, the findings cannot be generalized and needs to be validated in longitudinal studies. We are unable to elucidate the magnitude of the bidirectional relationship of prefrail and DM, and effect of DM with or without treatment on the trajectory of body composition. We did not have information on metformin use in pre-frail diabetics and if it conferred a protective role. The DM status is self-reported which may be subject to recall bias. Our study included only pre-frail participants; thus, our findings cannot be extrapolated to robust or frail populations. Despite the small sample size, the prevalence of self-reported DM was 51% and our study has generated interesting findings which requires further validation. Lastly, body composition measured by BIA may be affected by hydration, oedema and fasting but as our participants were community dwelling older adults, most of our participants were generally well and there was no fasting involved.

### Study Highlights and Future Directions

Our study further adds to the evolving literature on crossroad between frailty and diabetes where within gender, pre-frail older adults had similar body composition, cognition, and functional status. These findings should be explored in an external cohort for further validation. Both frailty and diabetes share a bi-directional relationship where frailty accelerates diabetes development, and diabetes accelerates frailty and ageing (22). Frailty is reversible, and predisposing factors for DM are modifiable with exercise and multidomain interventions (17, 59). While metformin has shown promising effect in extending health span, down regulating inflammation in diabetics and, its effects on delaying and reversing frailty and changing the trajectory of DM in pre-frail older adults is still an area of ongoing research (44). Longitudinal studies are needed to understand the magnitude and impact of chronic inflammation in both pre-frail older adults with and without DM, trajectory of DM development in non-diabetic pre-frail and progression of frailty and disability in pre-frail diabetics, and the role of metformin (55).

## Conclusions

There were insignificant physical function, cognition, and body composition differences within gender for pre-frail male and female except for higher prevalence of obesity defined by BMI and BF%, increase FMI and FFMI in females with DM. There were significant differences between gender for those with DM where females had overall lower muscle quality, lower physical function, cognition, perceived health with higher prevalence of depression. BMI, VFA >100, FMI and FFMI were found to be independently associated with DM status in pre-frail older adults. Further longitudinal studies are required to understand the pathogenesis, trajectory of DM and protective role of oral hypoglycemics in pre-frail older adults.

## Data Availability Statement

The raw data supporting the conclusions of this article will be made available by the authors, without undue reservation.

## Ethics Statement

The studies involving human participants were reviewed and approved by The National Healthcare Group Domain Specific Review Board (Reference: 2017/00035 and 2018/01183). The patients/participants provided their written informed consent to participate in this study.

## Author Contributions

RM: funding acquisition. RM and JM: methodology and conceptualization. RM and JS: formal analysis. RM: methodology and project administration. RM and JS: writing—original draft. RM, JS, and JM —review and editing. All authors contributed to the article and approved the submitted version.

## Funding

This study is part of a larger project that has been funded by Ministry of Health of Singapore: Healthy Ageing Innovation Grant under National Innovation Challenge on Active and Confident Ageing (Award No: MOH/NIC/HAIG02/2017) and National Medical Research Council (HSRG-HP17Jun003).

## Conflict of Interest

The authors declare that the research was conducted in the absence of any commercial or financial relationships that could be construed as a potential conflict of interest.

## Publisher’s Note

All claims expressed in this article are solely those of the authors and do not necessarily represent those of their affiliated organizations, or those of the publisher, the editors and the reviewers. Any product that may be evaluated in this article, or claim that may be made by its manufacturer, is not guaranteed or endorsed by the publisher.
